# Can COVID-19 Cause Flare-Ups of Acute Hepatitis B? An Atypical Presentation of COVID-19 with Acute Hepatitis B

**DOI:** 10.1155/2021/8818678

**Published:** 2021-02-04

**Authors:** Yavuz Yigit, Mahmoud Haddad, Amr Elmoheen, Mohamed Rezk Shogaa, Rabee Tawel, Y. Khatib Mohamed, Waleed Salem, Mahmoud Fawzy Eltawagny

**Affiliations:** ^1^Hamad Medical Corporation, Hamad General Hospital, Emergency Department, P. O. 3050, Doha, Qatar; ^2^Hamad Medical Corporation, Hamad General Hospital, Critical Care and Pulmonary Medicine, Doha, Qatar

## Abstract

We report a case of fulminant liver failure in a patient with acute active hepatitis B infection who was found to have COVID-19 without lung involvement. A 24-year-old male was brought by ambulance service to Hamad General Hospital, Emergency Department (ED), in Doha on April 8, 2020, with chief complaints of fever and cough for 3 days. Upon initial evaluation, the patient was febrile (39.4°C), jaundiced, and disoriented regarding time, place, and person, with an unremarkable past medical history. Initial blood tests showed severely elevated urea, creatinine, transaminases, and ammonium in addition to an impaired coagulation profile consistent with fulminant liver failure. A swab was taken for COVID-19 PCR testing and found to be positive. Serological tests revealed hepatitis B surface antigen positivity and other serology indicating acute hepatitis B. Initial X-ray and repeat chest X-rays did not show lung infiltrates. On the 6^th^ day after admission, the patient developed fixed dilated pupils, with brain edema on CT; cardiac arrest occurred on the 10^th^ day after admission, and the patient died. Although it is still largely unclear, HBV0-activated sudden-onset strong cytotoxic T lymphocyte response and enhanced viral replication and/or retention of the viral capsid in infected hepatocytes may cause the pathogenesis of FH. These pathophysiological events cause extensive hepatocyte apoptosis and necrosis, which results in deadly severe liver failure. Our findings support that the liver damage occurring in COVID-19 is caused by an impaired innate immune system rather than by direct cell damage caused by SARS-CoV-2. We think that more consideration should be given to the presence of acute hepatitis B, especially in COVID-19 patients.

## 1. Background

The association between COVID-19 and liver injury is defined as any liver damage that occurs during disease progression with or without preexisting liver disease. Approximately 14–53% of patients hospitalized with COVID-19 have elevated liver enzymes. To date, there has been no report of acute or acute-on-chronic liver failure in COVID-19 patients [1].

We report a case of fulminant liver failure in a patient with acute hepatitis B infection who was found to have COVID-19 without lung involvement.

## 2. Case Report

**Figure 1:**
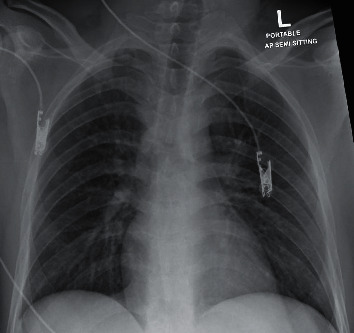
Chest X-ray on admission shows no lung infiltrates.

A 24-year-old male was brought by ambulance service to the ED with chief complaints of fever and cough for 3 days. Upon initial evaluation, the patient was febrile (39.4°C), jaundiced, and disoriented regarding time, place, and person, with an unremarkable past medical history. Physical examination revealed the following: GCS 14, blood pressure 126/84, heart rate 134/min, respiratory rate 23, and oxygen saturation 98% on room air. The examinations of the respiratory and cardiovascular systems were unremarkable. There was no sign of trauma or any findings of epileptic seizure disorder. A neurological examination revealed that cranial nerves II–XII were intact and symmetric; there were no meningeal signs, no abnormal skin findings, and no impairment in the movement of all four limbs. Other than jaundice, gastrointestinal exam was unremarkable. A venous blood gas test showed high anion gap metabolic acidosis with a lactate level of 22.8 mmol/L. The patient was transferred to the resuscitation area in the Emergency Department. Later, the patient became more confused and started to vomit, and he was intubated to secure the airway. Initial blood tests showed severely elevated urea, creatinine, and transaminases in addition to an impaired coagulation profile consistent with fulminant liver failure. Additionally, the ammonium levels were high and remained high for the course of the illness (Table 1). A chest X-ray (Figure 1) and head computed tomography (CT) were obtained and were unremarkable. A swab was taken for COVID-19 PCR testing and found to be positive (a second swab performed 5 days later was also positive). Due to the marked elevation of liver enzymes, which was not explained by COVID-19 alone, a presumptive diagnosis of accompanying causes, such as preexisting hepatitis or toxicologic causes, was considered, and relevant blood tests were performed. The serological tests revealed hepatitis B surface antigen positivity, and other serology indicated acute hepatitis B (Table 1). The acetaminophen level was elevated but in the therapeutic range (Table 1).

The patient underwent an urgent hemodialysis session. N-Acetylcysteine infusion, vitamin K, and fresh frozen plasma were given. The patient was transfused with intravenous crystalloids and started on a proton-pump inhibitor infusion. A COVID-19 treatment protocol was initiated with azithromycin, chloroquine, and oseltamivir.

**Figure 2:**
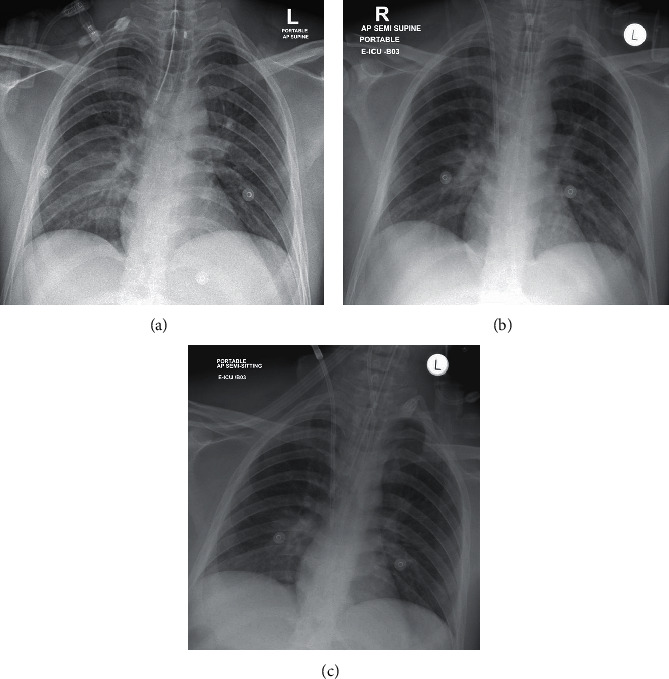
Repeat chest X-rays also did not show any classical findings for COVID-19.

**Figure 3:**
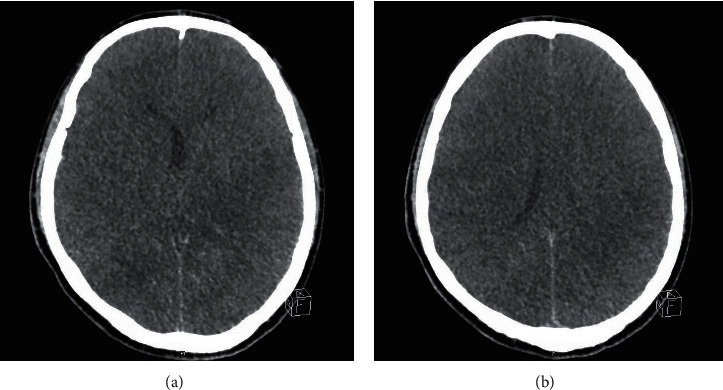
CT head slides showing brain edema on the 6^th^ day of admission.

The patient was admitted to the intensive care unit where artificial ventilation was continued. Repeat chest X-rays did not show lung infiltrates (Figure 2). The patient required multiple sessions of hemodialysis. The severe metabolic acidosis and drastically high liver enzyme levels improved, and the patient was removed from sedation but did not wake up. A diagnosis of hepatic encephalopathy was considered in view of the liver failure and persistently high ammonia levels despite treatment. An ultrasound of the abdomen was ordered to differentiate acute versus chronic liver disease and to guide therapy. The ultrasound showed a normally sized liver with a slightly coarse texture, without cirrhotic features. On the 6^th^ day after admission, the patient developed fixed dilated pupils, with brain edema on CT (Figure 3) and CT Head Video; despite maximal support, he went into cardiac arrest on the 10^th^ day after admission and died.

No written consent has been obtained from the patient as there are no patient identifiable data included in this case report.

## 3. Discussion

In this case report, we present a 24-year-old patient who experienced acute hepatitis B and atypical COVID-19 at the same time and died in the hospital as a result of a very severe and rapid course of illness. To the best of our knowledge, to date, there are no cases in the literature of COVID-19 accompanied by acute hepatitis B, resulting in fulminant hepatitis [1]. We think that COVID-19 combined with acute hepatitis B may have caused the disease to progress to fulminant hepatitis.

Most symptomatic SARS-CoV-2 infections are mild to moderate, not severe. A report from the Chinese Center for Disease Control and Prevention [2] of approximately 44,500 patients with confirmed infections shows the following disease severity factors:

- 81 percent had mild disease (without or with mild pneumonia)
- 14 percent had severe disease (with dyspnea, hypoxia, or >50 percent affected lung fields on imaging within 24 to 48 hours)
- 5 percent had critical disease (e.g., with respiratory failure, shock, or multiorgan dysfunction)
- A case fatality rate of 2.3 percent, with no deaths occurring in noncritical cases

The most common serious manifestation is pneumonia, which manifests primarily with fever, cough, and dyspnea and with bilateral infiltrates on pulmonary imaging [3–6]. Sore throat, rhinorrhea, and headache are other less common symptoms. During the early or mild disease period, normal chest radiographs can be seen. Consolidation and ground glass opacities with bilateral, peripheral, and lower lung field distributions are the most common imaging findings. During the course of the disease, the lung involvement increases, and on days 10 to 12 after symptom onset, a peak in the severity of lung involvement can be seen [7]. None of the classical lung imaging findings of COVID-19 occurred in our patient in the 15 days from hospitalization to death.

ARDS, which affects 20%–41% of hospitalized patients, is a major complication of COVID-19 [3, 8]. Neutrophilia and elevated lactate dehydrogenase and D-dimer levels have been reported as risk factors for ARDS and death in COVID-19 patients [8]. Although MODS developed shortly after hospitalization and LDH and D-dimer levels were high at and after admission, our patient did not develop ARDS.

Our laboratory findings are compatible with an exuberant inflammatory response with elevated inflammatory markers (e.g., D-dimer and ferritin), similar to cytokine release syndrome in severe COVID-19 cases. Despite this severe inflammatory response, the absence of ARDS and the classical lung imaging findings of COVID-19 is an atypical course.

For most cases, acute hepatitis B is a self-limiting disease that lasts for several weeks [9]. In most cases, patients need only supportive treatment. Elderly patients, patients who have preexisting liver disease, immunocompromised patients, and patients who have concomitant infection with hepatitis C or human immunodeficiency virus (HCV or HIV) have a relatively worse prognosis [10]. Our patient had none of these risk factors. Fulminant hepatitis (FH) is defined as an INR >1.5, with the presence of hepatic encephalopathy but the absence of chronic, underlying (or prior) disease [11]. Approximately 0.1% to 0.5% of all patients develop FH [10]. HBeAg negativity, age over 34 years old, and total bilirubin over 10 mg/dl are independent risk factors for FH B [12]. Again, none of these risk factors were present in our patient.

A superinfection with another hepatotropic virus, such as hepatitis A, C, E, or delta, can cause a flare-up of chronic HBV infection; therefore, for suspected flares in chronic HBV infection patients, these viruses should always be ruled out. Some studies report higher mortality rates in the case of superinfection with HAV [13] and that the risk of HBV reactivation was greater in the event of superinfection with HEV [14]. Superinfection with the delta virus may cause a severe exacerbation of HBV. Our patient's blood tests, ultrasound, and past medical history did not show any sign of an underlying chronic disease or any concomitant infection other than COVID-19.

The reasons for the development of such aggressive FH in a young healthy patient without any risk factors for healthy FH are important. The pathogenesis of FH is largely unclear. A sudden-onset strong cytotoxic T lymphocyte response due to HBV, enhanced viral replication, and/or retention of the viral capsid in infected hepatocytes are blamed for the pathophysiology [15, 16]. The result of these pathophysiological events is extensive hepatocyte apoptosis and necrosis, leading to an immense loss of liver function that is deadly in HBV-infected individuals. We think that COVID-19 may have played a facilitating role in the formation of this extreme inflammatory response in our patient.

SARS-CoV-2 shares an 82% genome sequence similarity with SARS-CoV and 50% genome sequence homology to Middle East respiratory syndrome coronavirus (MERS-CoV); all three coronaviruses are known to cause severe respiratory symptoms [17].

Up to 60% of SARS patients are reported to have liver impairment [18], and MERS-CoV patients are also reported to have liver impairment. Abnormal levels of chemokines and cytokines have been detected during the early stages of SARS-CoV infection. Duan et al. [19] reported higher IL-1, IL-6, and IL-10 levels in SARS patients with abnormal liver function compared to normal patients and suggested that the inflammatory response was the cause of the liver damage. Additionally, Huang et al. [20] found that SARS patients are more likely to develop liver injury and severe hepatitis when they have an accompanying HBV infection.

Bangash et al. [21] suggested that virally induced cytotoxic T cells and induction of a dysregulated innate immune response were the causes of abnormal liver markers in COVID-19 patients, which is a similar mechanism observed in FH. Normally, the liver encounters and filters many different kinds of exogenous material, and it maintains immune tolerance through the gut-liver axis. However, in COVID-19 patients, physiological stress conditions can cause interruptions in immune tolerance. The hyperactivated immune response and systemic inflammation caused by the cytokine storm can cause liver damage. Indeed, the liver is the most frequently damaged organ outside the respiratory system in COVID-19 patients [22]. Studies have shown that, in severe COVID-19 patients, serum levels of Th17 and CD8 T cells, tumor necrosis factor-a, granulocyte-colony stimulating factor, interferon inducible protein-10, monocyte chemotactic protein 1, macrophage inflammatory protein 1 alpha, interleukin-2, interleukin-6, interleukin-7, and interleukin-10 are significantly higher than those in control patients [22].

## 4. Conclusions

To date, no acute or acute-on-chronic liver failure has been documented in COVID-19 patients, and there is no report of death directly related to hepatic decompensation in patients without preexisting liver disease [1]. This is the first case in the literature of FH that developed in the presence of COVID-19 accompanying acute hepatitis B. Our findings support that the liver damage of COVID-19 is caused by an impaired innate immune system rather than by direct cell damage caused by SARS-CoV-2. We think that more consideration should be given to the presence of acute hepatitis B, especially in COVID-19 patients.

## Figures and Tables

**Table 1:** Clinical laboratory results on days 0, 3, and 6 of treatment.

Item	Day 0	Day 3	Day 6	Reference range
Body temperature (°C)	39.4	36	39.9	36.1–37.0
Pulse (beats/min)	134	85	96	60–100
Respiratory rate (breaths/min)	27	MV	MV	12–20
Blood pressure (mmHg)	126/84	136/85	150/93	90–140/60–90
Hemoglobin (g/dL)	13.9	9.1	9.8	13–17
White blood cell (×10^3^/*μ*L)	10	8.1	10.1	04–10
Neutrophil (%)	78	56.5	60	50–75
Absolute neutrophil (×10^3^/*μ*L)	8	4.6	6.1	2.0–7.5
Lymphocyte (%)	14	26.3	16	20–40
Absolute lymphocyte (×10^3^/*μ*L)	1.4	2.1	1.6	0.8–4.0
Platelets (×10^3^/*μ*L)	64	32	39	150–400
C-reactive protein (mg/L)	67.4	11.8	<5	0–10
Procalcitonin (ng/mL)	0.8	0.81	0.71	0–0.5
Lactate (mmol/L)	22.8	7	6.7	0.36–1.6
pH value	7.14	7.5	7.53	7.350–7.450
Oxygen saturation (%)	98	100	100	94–100
Bicarbonate (mmol/L)	7.4	25	25	22–29
Alanine aminotransferase (U/L)	>7000	1089	188	0–40
Aspartate aminotransferase (U/L)	>7000	526	98	0–41
Total bilirubin (*μ*mol/L)	115	134	210	0–21
Direct bilirubin (*μ*mol/L)	66	49.4	65.5	0–5
Alkaline phosphatase (U/L)	174	139	132	40–129
Creatinine (*μ*mol/L)	305	635	568	62–106
Glucose (mmol/L)	3.2	8.5	13.7	3.9–6.1
Ammonia (*μ*mol/L)	268	265	162	16–60
D-Dimer (mg/L)	11.5	8.7	33.18	0–0.5
Fibrinogen (g/L)	1.75	1.5	0.4	2–4.1
INR	>10	7	2.2	<1.1
PT (seconds)	>120	84.1	26.6	9.4–12.5
APTT (seconds)	64	44.9	40.3	25.1–36.5
Ferritin (*μ*g/L)	20573	2984	1447	38–270
Ethanol	Negative			
Salicylate	Negative			
Blood culture and sensitivity	Negative		Negative	
Hepatitis B core Ab	Reactive			
Hepatitis B core Ab IgM	Reactive			
Hepatitis B surface antigen	Reactive			
Hepatitis B surface Ab	Nonreactive			
Hepatitis B surface Ab Num.	<2.00			
Hepatitis Be antigen	Reactive			
Hepatitis Be Ab	Nonreactive			
Hepatitis C Ab	Nonreactive			
Hepatitis B D Ab	Nonreactive			
Hepatitis total A Ab	Reactive			

## Data Availability

The data used to support the findings of this study are included within the article.
